# Symptomatic Improvement Following Intra-articular Injection of Cultured Autologous Adipose-Derived Mesenchymal Stem Cells in Kellgren-Lawrence Grade 3 Knee Osteoarthritis: A Case Report With One-Year Follow-Up

**DOI:** 10.7759/cureus.113830

**Published:** 2026-08-02

**Authors:** Yuichi Wakabayashi

**Affiliations:** 1 Regenerative Medicine, Cell Grand Clinic, Osaka, JPN

**Keywords:** adipose-derived mesenchymal stem cells, cartilage regeneration, case report, intra-articular injection, knee osteoarthritis, meniscus, regenerative medicine

## Abstract

Knee osteoarthritis (OA) is a progressive joint disease, and treatment options for cases refractory to conservative therapy can be limited. We present a 67-year-old woman with Kellgren-Lawrence (KL) grade 3 OA of the left knee who received an intra-articular injection of cultured autologous adipose-derived mesenchymal stem cells (ADMSCs) after conservative measures failed to provide durable relief. She had experienced progressively worsening left knee pain over approximately one year; intra-articular hyaluronic acid and platelet-rich plasma (PRP) injections had produced only transient benefit. ADMSCs cultured from autologous abdominal adipose tissue (100 million cells, 97.92% viability) were injected into the left knee joint under ultrasound guidance on Day 1, in accordance with a plan filed under Japan's Act on the Safety of Regenerative Medicine (plan number PB5240089). The pain visual analogue scale (VAS) improved from 8/10 at baseline to 1/10 at approximately 2.5 months and 0/10 at approximately 13 months. The Japanese Orthopaedic Association knee OA treatment score rose from 55/100 at baseline to 90/100 at approximately 2.5 months and 95/100 at approximately 13 months. Joint effusion resolved, and range of motion and gait improved. Serial MRI over the follow-up period showed improvement of the lateral meniscal lesion and an increase in tibiofemoral cartilage thickness. No serious adverse events occurred. As a single, uncontrolled case, causal inference and generalizability are limited. Nonetheless, intra-articular ADMSC injection may be a viable option for conservative-therapy-refractory knee OA and warrants controlled prospective evaluation.

## Introduction

Knee osteoarthritis (OA) is a progressive disease characterized by degeneration of articular cartilage, subchondral bone changes, and chronic intra-articular inflammation associated with aging and mechanical loading [1]. In moderate-to-advanced disease that remains symptomatic despite conservative measures, such as exercise therapy, analgesics, and intra-articular hyaluronic acid, total knee arthroplasty is a well-established option; however, some patients decline surgery or hospitalization [2]. Against this background, interest has grown in regenerative approaches aimed at symptom relief and improvement of the joint tissue environment.

Intra-articular injection of mesenchymal stem cells (MSCs), and adipose-derived MSCs (ADMSCs) in particular, has been reported in several randomized controlled trials to improve pain and function in knee OA with a favorable safety profile [3-6]. Rather than direct cartilage regeneration, the mechanism is thought to be predominantly paracrine, mediated by anti-inflammatory activity, trophic factor secretion promoting tissue repair, and immunomodulation [7]. Evidence also suggests that repeated dosing may yield better outcomes than a single dose [8]. Reported outcomes across randomized trials have been heterogeneous, with some analyses favoring adipose-derived cells and higher cell doses [9] while others describe only small average benefits of limited certainty [10]. This heterogeneity underscores the current evidence gap and the value of carefully reported individual clinical courses delivered within a regulatory framework.

Here, we present a 67-year-old woman with Kellgren-Lawrence (KL) grade 3 OA of the left knee and a lateral meniscus tear who had not obtained durable benefit from conservative therapy, and who was treated with intra-articular cultured ADMSCs, with one-year follow-up. Cultured autologous ADMSCs were selected to provide a defined, characterized cell product derived from the patient's own tissue, avoiding the immunogenicity and pooling concerns of allogeneic sources, while acknowledging that comparative superiority over other regenerative options is not established. We report the clinical course while explicitly acknowledging the limitations inherent to a single, uncontrolled case.

## Case presentation

A 67-year-old right-handed woman working as a clerk (height 171 cm, weight 65 kg, body mass index (BMI) 22.2) presented with left knee pain. She had noticed left knee discomfort approximately one year earlier, which progressed to pain on stair climbing and first-step pain on rising in the morning.

Her medical history included ulcerative colitis, cervical spondylosis, hand OA (surgically treated approximately a decade earlier), and atrial fibrillation. She took no regular medications and had no drug or food allergies. She was a non-smoker and consumed alcohol twice weekly. Her mother had OA. She had been diagnosed with left knee OA at another facility, where she received a single intra-articular injection of hyaluronic acid (transient effect) and, subsequently, a single intra-articular injection of leukocyte-rich platelet-rich plasma (effect lost within about one month). The interval between the platelet-rich plasma injection and the ADMSC injection was approximately four months. She declined total knee arthroplasty and hospitalization because of personal preference and reluctance to undergo surgery while remaining occupationally active, and presented to our clinic. She did not undergo any structured rehabilitation or supervised exercise therapy during the subsequent follow-up period.

At the initial visit (Day -42), left knee pain was 8/10 on the visual analogue scale (VAS) (0-10 scale). Examination revealed a positive patellar tap with joint effusion (approximately 10 mL) and neutral (normal) lower-limb alignment. There was tenderness over the lateral joint line and intermittent gait limitation (able to walk about 100 m but with difficulty at 300 m). The McMurray test was negative. Knee range of motion was restricted (extension -10 degrees, flexion 120 degrees).

On comorbidity assessment, blood tests showed no overt diabetes (HbA1c 5.4%) but dyslipidemia (total cholesterol 231 mg/dL, low-density lipoprotein (LDL) 119 mg/dL). Hepatic and renal function and complete blood count were essentially normal. She had no pacemaker or intracorporeal metal and was independent in activities of daily living.

**Figure 1:**
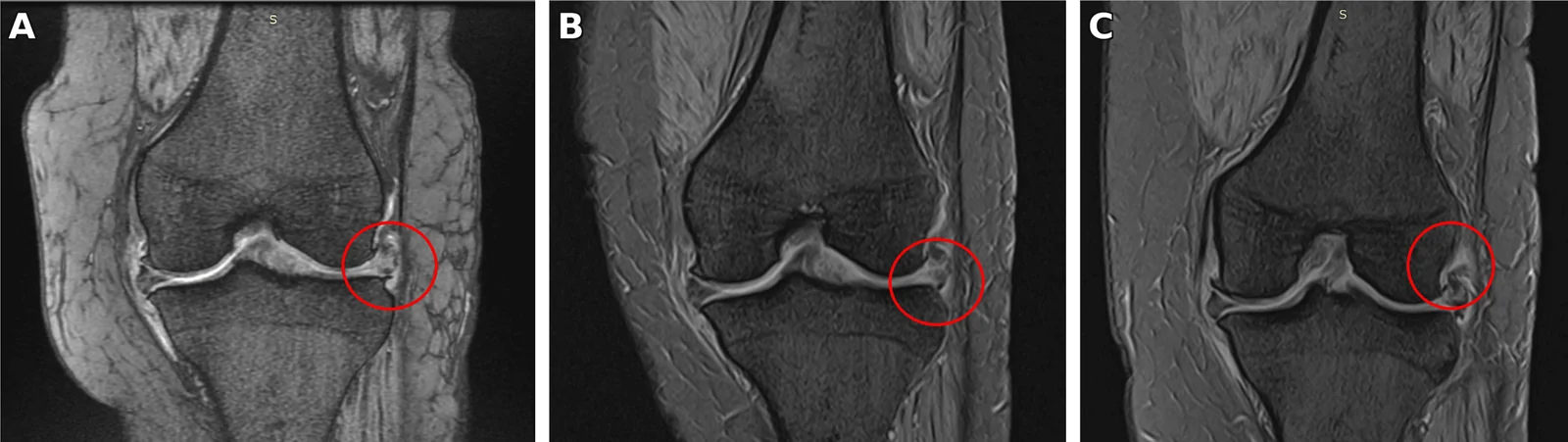
Serial coronal T2*-weighted MRI of the left knee before and after intra-articular ADMSC injection Serial coronal T2*-weighted MRI of the left knee. (A) Pretreatment (Day -52); (B) Day 78; (C) Day 407. Red circles indicate the lateral meniscal lesion, which improved in signal and morphology over time. Panel A was acquired on a different scanner. ADMSC: adipose-derived mesenchymal stem cell

**Figure 2:**
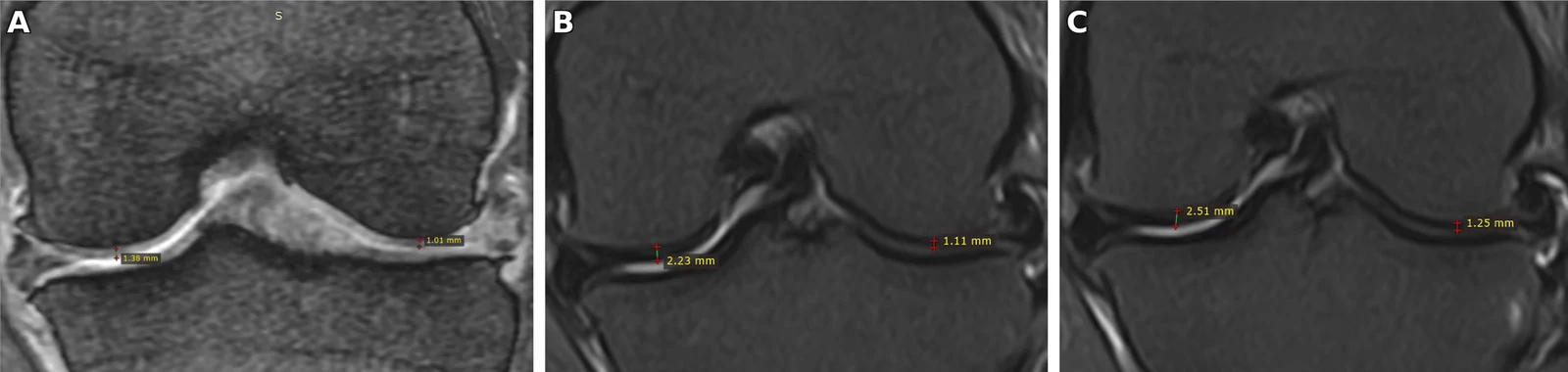
Serial coronal MRI of the left knee showing tibiofemoral cartilage-thickness measurements Serial coronal MRI of the left knee showing tibiofemoral cartilage-thickness measurements. (A) Day -52; (B) Day 78; (C) Day 407. Cartilage thickness increased at the two measured points (1.38 to 2.23 to 2.51 mm and 1.01 to 1.11 to 1.25 mm). Panel A was acquired on a different scanner without a T2 STIR sequence. Cartilage thickness was measured using the anterior cruciate ligament as the anatomical landmark to approximate the same location across time points; measurements were made by a single, non-blinded assessor without formal reliability testing. STIR, short tau inversion recovery

Imaging showed KL grade 3 on plain radiographs. Knee MRI was obtained before treatment (Day -52) and after treatment (Day 78 and Day 407). The pretreatment MRI showed lateral meniscal injury/degeneration on T2*-weighted images and lateral joint-space narrowing, and the patient was diagnosed with left OA (KL grade 3) with a lateral meniscus tear. On serial comparison, the lateral meniscal lesion improved in signal and morphology (Figure 1), and tibiofemoral cartilage thickness increased at the measured points (from 1.38 to 2.23 to 2.51 mm at one site and from 1.01 to 1.11 to 1.25 mm at the other; Figure 2). Of note, only the pretreatment scan was obtained on a different scanner (without a T2 short tau inversion recovery (STIR) sequence), which limits strict cross-scanner quantitative comparison.

**Figure 3:**
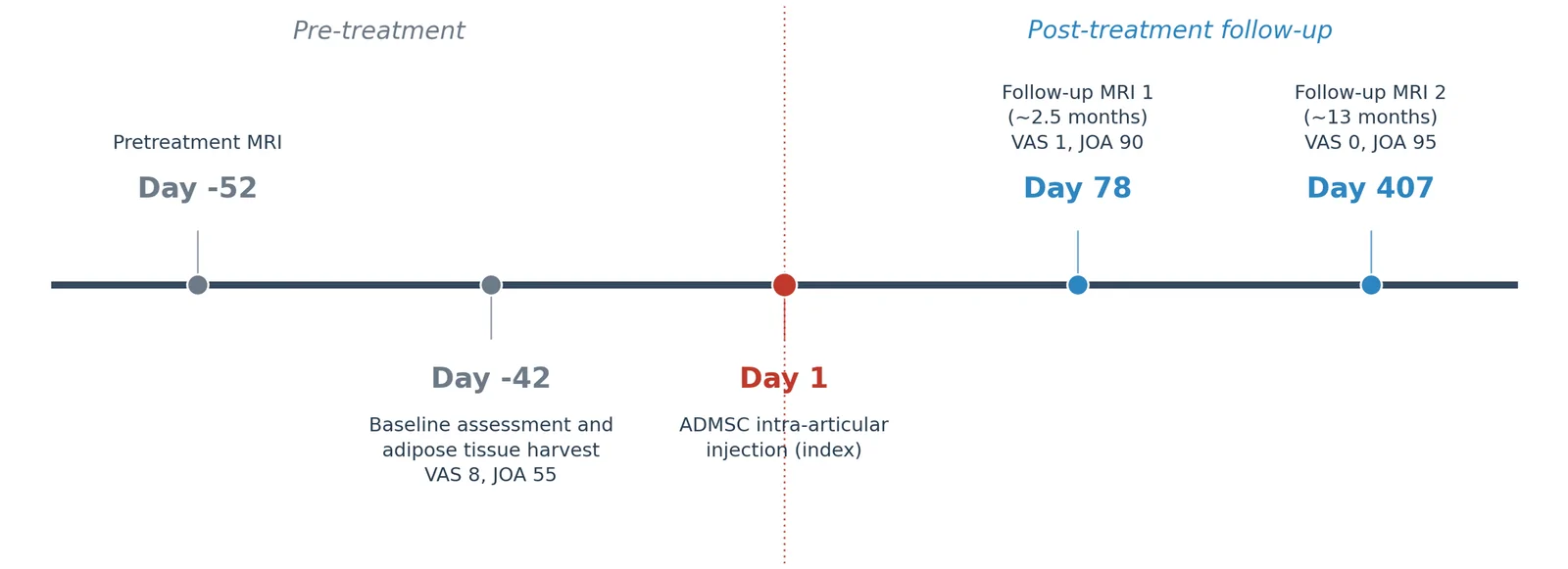
Graphical timeline of the clinical course Graphical timeline of the clinical course. Day 1 denotes the day of intra-articular ADMSC injection (index treatment); negative days precede treatment. ADMSC: adipose-derived mesenchymal stem cell; VAS: visual analogue scale; JOA: Japanese Orthopaedic Association

The overall clinical course is summarized in Figure 3. Regarding treatment, on Day -42, on the same day as the baseline assessment, approximately 5 mL of autologous adipose tissue was harvested from the abdomen. ADMSCs cultured from this tissue were administered at 1×10⁸ cells, with 97.92% viability, passage number 3, and a culture period of approximately five weeks. The interval from adipose harvest to intra-articular injection was approximately six weeks, as the injection was scheduled at the patient's convenience. The cells were processed at a licensed cell-processing center and met International Society for Cellular Therapy (ISCT) criteria: by flow cytometry, they were positive for CD73 (99%), CD90 (98%), and CD105 (99%), and negative for the hematopoietic and endothelial markers CD34, CD45, CD11b, CD19, and HLA-DR. Trilineage (osteogenic, chondrogenic, and adipogenic) differentiation capacity was established during facility-level validation of the protocol and was not reassessed for this individual lot. Each product was derived exclusively from the patient's own adipose tissue, with no batch pooling. Release testing confirmed sterility (bacterial culture negative (<1 colony), endotoxin <0.3 EU/mL, and mycoplasma negative), and the product was stored refrigerated (not cryopreserved). On Day 1, approximately 2 mL of ADMSCs was injected into the left knee joint under ultrasound guidance (the index treatment). The treatment was carried out under a regenerative medicine provision plan filed in accordance with Japan's Act on the Safety of Regenerative Medicine (plan number PB5240089).

**Figure 4:**
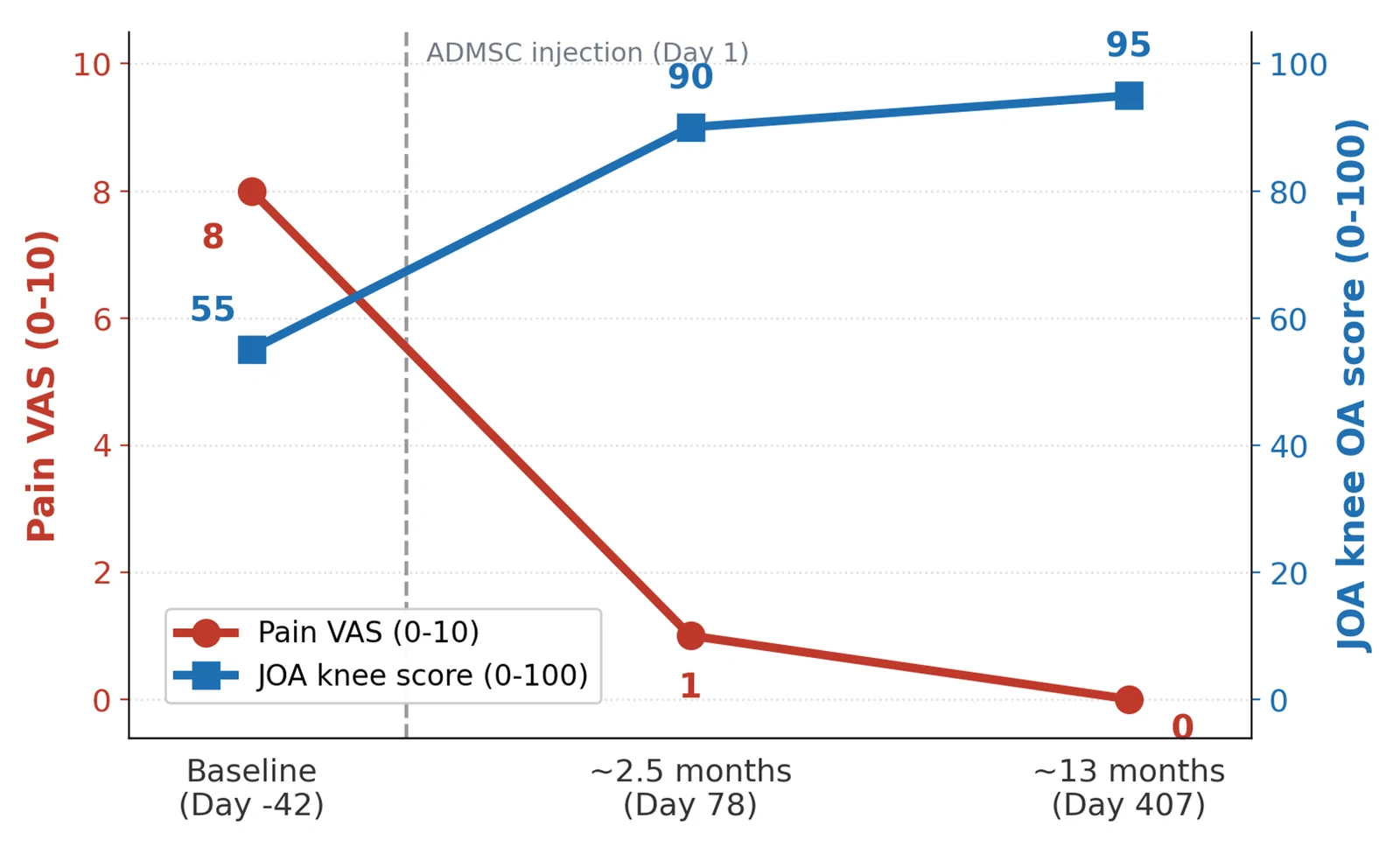
Serial pain VAS and JOA knee osteoarthritis score over time Serial pain VAS (0-10) and JOA knee osteoarthritis score (0-100) from baseline (Day -42) through Day 407 (approximately 13 months). The pain VAS decreased from 8 to 1 to 0, and the JOA score increased from 55 to 90 to 95. JOA: Japanese Orthopaedic Association; VAS: visual analogue scale

Outcomes were as follows. The serial pain VAS and JOA scores are shown in Figure 4. The pain VAS (0-10) improved from 8 at baseline (Day -42) to 1 at the first follow-up (Day 78, ~2.5 months) and 0 at the final follow-up (Day 407, ~13 months). The Japanese Orthopaedic Association knee OA treatment score (0-100, physician-rated) improved from 55/100 at baseline (Day -42) to 90/100 at the first follow-up (Day 78) and 95/100 at the final follow-up (Day 407). On examination, joint effusion resolved and range of motion and gait improved; the patient reported being pain-free with increased activity. The only adverse events were minor: transient bruising at the adipose harvest site and transient tightness after intra-articular injection; no other or serious adverse events occurred.

## Discussion

This case describes a 67-year-old woman with conservative-therapy-refractory KL grade 3 OA who underwent intra-articular injection of cultured ADMSCs, with improvement in pain and functional measures over one year. The VAS improved from 8 to 0 and the Japanese Orthopaedic Association score from 55 to 95, accompanied by objective changes such as resolution of joint effusion, improved gait, and serial MRI changes (Figures 1-2).

The efficacy and safety of intra-articular ADMSC injection for knee OA have been reported in several randomized controlled trials [3-6], and the present course is consistent with these reports. MSC activity is thought to be predominantly paracrine, mediated by anti-inflammatory effects, trophic factor secretion, and immunomodulation, rather than direct cartilage regeneration [7], and the resolution of effusion and pain relief may reflect such changes in the intra-articular environment. This patient had a concurrent lateral meniscus tear; it has been reported that synovial-fluid MSCs increase after meniscus injury [11] and that MSC transplantation can promote meniscus regeneration [12], so involvement of meniscal pathology is theoretically plausible. In this case, serial MRI suggested improvement of the lateral meniscal lesion (Figure 1) and an increase in tibiofemoral cartilage thickness (Figure 2); however, imaging alone cannot establish true tissue regeneration, and the different baseline scanners are a caveat. Accordingly, the cartilage-thickness observations are regarded as descriptive only and do not by themselves indicate structural cartilage regeneration.

This report has important limitations. First, as a single case without a control group, the observed improvement cannot be attributed to ADMSC therapy alone. Natural fluctuation of OA symptoms, regression to the mean, a placebo or expectation effect, and delayed effects of the prior intra-articular hyaluronic acid and platelet-rich plasma injections are all plausible contributors that cannot be excluded. Consistent with this caution, a recent GRADE-based meta-analysis reported that intra-articular MSC therapy probably yields little-to-no average improvement in pain or physical function at the group level [10], underscoring that a single favorable course must be interpreted conservatively. Second, follow-up was one year, so long-term durability and the potential need for repeat dosing (which some reports suggest may yield better outcomes [8]) could not be evaluated. Third, the Japanese Orthopaedic Association score is physician-rated, introducing potential assessor bias. Fourth, the pretreatment MRI was obtained on a different scanner without a T2 STIR sequence, so the serial cartilage-thickness measurements should be interpreted with caution given cross-scanner variability.

Given these limitations, this case does not establish causation and constitutes a descriptive account of the clinical course in a single patient. Nonetheless, it may be of value to present the course of regenerative treatment, performed within a regulatory framework, as one option for patients with conservative-therapy-refractory knee OA who decline surgery. Prospective studies with appropriate controls and long-term follow-up are needed to verify the contribution of each component and the durability of the effect.

## Conclusions

In a 67-year-old woman with conservative-therapy-refractory KL grade 3 OA, intra-articular injection of cultured autologous ADMSCs was associated with improvement in pain and functional measures over one year, with no serious adverse events. However, because this is a single, uncontrolled case and because follow-up duration and objective measures are limited, causation cannot be concluded. This is a hypothesis-generating report that warrants verification through controlled prospective studies.
